# Dental pulp-derived stem cell conditioned medium to regenerate peripheral nerves in a novel animal model of dysphagia

**DOI:** 10.1371/journal.pone.0208938

**Published:** 2018-12-11

**Authors:** Takeshi Tsuruta, Kiyoshi Sakai, Junna Watanabe, Wataru Katagiri, Hideharu Hibi

**Affiliations:** 1 Department of Oral and Maxillofacial Surgery, Nagoya University Graduate School of Medicine, Nagoya, Japan; 2 Division of Reconstructive Surgery for Oral and Maxillofacial Region, Niigata University Graduate School of Medical and Dental Sciences, Niigata, Japan; Università degli Studi della Campania, ITALY

## Abstract

In nerve regeneration studies, various animal models are used to assess nerve regeneration. However, because of the difficulties in functional nerve assessment, a visceral nerve injury model is yet to be established. The superior laryngeal nerve (SLN) plays an essential role in swallowing. Although a treatment for SLN injury following trauma and surgery is desirable, no such treatment is reported in the literature. We recently reported that stem cells derived from human exfoliated deciduous teeth (SHED) have a therapeutic effect on various tissues via macrophage polarization. Here, we established a novel animal model of SLN injury. Our model was characterized as having weight loss and drinking behavior changes. In addition, the SLN lesion caused a delay in the onset of the swallowing reflex and gain of laryngeal residue in the pharynx. Systemic administration of SHED-conditioned media (SHED-CM) promoted functional recovery of the SLN and significantly promoted axonal regeneration by converting of macrophages to the anti-inflammatory M2 phenotype. In addition, SHED-CM enhanced new blood vessel formation at the injury site. Our data suggest that the administration of SHED-CM may provide therapeutic benefits for SLN injury.

## Introduction

Peripheral nerve injury following trauma and surgery is a severe clinical problem that results in potential long-term disability and a reduction in the patient’s quality of life. In peripheral nerve regeneration studies, the sciatic, facial, femoral and median nerves in rodents and other larger animals are widely used to assess nerve regeneration [1]. For clinical application, nerve-specific evaluation following several types of nerve injury is necessary, but there are no previous reports on visceral nerve regeneration. Thus, an animal model of visceral nerve lesion has not been established owing to difficulties in assessing quantitative nerve function.

The superior laryngeal nerve (SLN) originates from the vagus nerve and plays an important role in swallowing [2]. The SLN is a visceral sensory nerve that supplies the pharyngeal and supraglottic mucosa [3]. Injury to the SLN during surgery, such as neck dissection, thyroidectomy, anterior approaches to the cervical spine, or carotid endarterectomy, causes dysphagia and subsequent aspiration pneumonia due to sensory loss of the laryngopharynx and a reduction in the force of glottis closure [4]. Treatment of SLN injury is not reported in the literature. Therefore, the development of effective therapies for patients with dysphagia following SLN injury is necessary. However, there are no reports regarding SLN regeneration owing to the lack of an evaluable experimental animal model.

In recent years, researchers started investigating stem cell-based transplantation therapy as a promising strategy for tissue regeneration. Stem cells from human exfoliated deciduous teeth (SHEDs) and human dental pulp stem cells (hDPSCs) are self-renewing mesenchymal stem cells derived from the perivascular niche of the dental pulp [5]. They are thought to originate from the cranial neural crest that expresses early mesenchymal and neuroectodermal stem cell markers [6–9]. They are able to maintain stemness properties in 3D culture [10]. These stem cells are relatively easy to collect and exhibit high plasticity and multi-potential capabilities [7]. We have previously shown that SHEDs and hDPSCs transplantation in spinal cord injury promote the functional recovery of hind limb movement [11]. Engrafting SHEDs facilitates successful peripheral nerve and central nervous system regeneration in a paracrine fashion, activating intrinsic tissue-repairing activities [12–14]. Transplantation of hDPSCs enhanced angiogenesis in sciatic nerve resection and part of the stem cells differentiated into nerve cells [15, 16]. Our studies have also shown that serum-free cultured conditioned medium (CM) from SHEDs (SHED-CM) contains various factors that promote functional recovery after peripheral nerve and central nervous system injury [17, 18]. Administration of CM avoids the disadvantages of cell transplantation, such as tumorigenesis, strong immune reactions and the difficulty in having a stable supply of cells. Furthermore, we have recently shown that the SHED-CM promotes tissue regeneration by converting the macrophage phenotype from pro-inflammatory M1 macrophages, which accelerate tissue destruction, to anti-inflammatory M2 macrophages, which promote tissue repair [19].

The objective of this study was to validate a novel nerve injury model of SLN lesion in the rat. Furthermore, we examined the therapeutic effects of intravenous administration of SHED-CM in this model.

## Materials and methods

### Study approval

All experimental procedures involving animals were conducted in accordance with the National Institutes of Health Guidelines for the Care and Use of Laboratory Animals, and were approved by the Nagoya University School of Medicine Animal Care and Use Committee. Exfoliated deciduous teeth from humans were collected under the guidelines approved by Nagoya University (2015–0278). Ethical approval was obtained from the Ethics Committee of Nagoya University (permission number 8–2). We obtained written informed consent from all patients; in the case of minors, written informed consent was given by their parents or guardians.

### Isolation of SHEDs and cell culture

SHEDs were isolated as described previously [9]. In brief, human exfoliated deciduous teeth were collected from patients aged 6–12 years. The dental pulp was separated from the crown and root of the tooth. The isolated pulp was subsequently digested in a solution of 3 mg/mL of collagenase type I and 4 mg/mL of dispase for 1 h at 37 °C. Single-cell suspensions were cultured in Dulbecco’s Modified Eagles’ Medium (DMEM) (Gibco, Rockville, MD) supplemented with 10% fetal bovine serum and with an antibiotic-antimycotic solution (100 units/mL penicillin G, 100 mg/mL streptomycin, and 0.25 mg/mL amphotericin B; Gibco) and incubated at 37 °C in an atmosphere of 5% CO_2_/ 95% air. SHEDs used in this study exhibited a fibroblastic morphology with a bipolar spindle shape, expressed MSC markers (CD90, CD73 and CD105) but not endothelial/hematopoietic markers (CD34, CD45, CD11b/c or HLA-DR), and were capable of undergoing adipogenic, chondrogenic and osteogenic differentiation [11].

### Preparation of CM

After the SHEDs reached 80% confluency, the medium was replaced with serum-free DMEM (DMEM (-)) containing the antibiotic-antimycotic solution. The cell-culture’s CM was collected after a 48-h incubation period. The CM was collected by centrifugation for 5 min at 440 × g, and was centrifuged again for 3 min at 17,400 × g to remove cell debris. We used the SHED-CM without either enrichment or dilution. The CM was collected and stored at 4 °C before use in the following experiments.

### Animals

All animal experiments undertaken in this study were performed in strict accordance with the protocols approved by the Institutional Animal Care Committee. Male Wistar/ST rats weighing 300–330 g (9–10 weeks old) were obtained from Japan SLC Shizuoka, Japan Inc. All rats were maintained on a 12-h light/ dark cycle with free access to food and water.

### Surgical procedures

All rats were anesthetized using an intraperitoneal (i.p.) administration of a mixture of medetomidine (0.15 mg/kg, i.p.; Domitor; Nippon Zenyaku Kogyo Co., Ltd., Fukushima, Japan), midazolam (2 mg/kg, i.p.; Dormicum Astellas Pharma Inc., Tokyo, Japan) and butorphanol (2.5 mg/kg, i.p: Vetporphale Meiji Seika Pharma CO., Ltd., Tokyo, Japan). Anesthetized rats were maintained at a constant temperature of 37 °C on a warming plate. The neck skin was shaved and opened under a surgical microscope (Olympus, Tokyo, Japan). The SLN was exposed bilaterally and injured with a vascular clip (60 g/mm^2^; NATSUME SEISAKUSHO Co Ltd., Tokyo, Japan) over a period of 30 min. The muscle and skin layers were closed with 4–0 Vicryl sutures (Ethicon Inc., Somerville, NJ). The animals were randomly assigned to the following four groups: (1) Sham: SLN exposure without any damage to the nerve tissue; (2) Injury: not injected; (3) DMEM (-): 1 ml DMEM (-)-injected into the tail vein for 10 s simultaneously with the SLN damage; (4) SHED-CM: 1 ml SHED-CM-injected into the tail vein for 10 s simultaneously with the SLN damage.

### Measurements of food intake and body-weight

Following SLN injury, the body-weight and food intake of rats were measured daily. Animals had free access to food and water.

### Swallowing analysis

Two weeks before the swallowing analysis, rats were placed in a custom-designed plastic test cage (300 × 150 × 200 mm) twice per week for 2 h with free access to water in a water bottle inclined at about 45 degrees, which was located 75 mm above the cage bottom. At 7 days after surgery, after 16-h of water restriction, which was reported to induce thirst [20, 21], each animal was placed in a test cage with free access to water. Swallowing was recorded using a digital video camera (HDR-AS50; Sony, Tokyo, Japan), and the volume of water intake was measured for 2 min. The videos were recorded at 120 frames per second and at a resolution of 1280 × 720. Video images were digitized for frame-by-frame analysis using movie analysis software (PowerDirector 15; Cyberlink, Tokyo, Japan), and the number of water intake interruptions and lick rate (lick cycle per second) were measured. Lick rate started to be counted the first time the tongue was maximally protruded at the spout, and each subsequent maximal tongue protrusion was counted [20].

### Measurement of the swallowing reflex

The method used for measuring the swallowing reflex was previously reported [3, 22, 23]. Briefly, at 7 days after surgery, the swallowing reflex was elicited experimentally by intra-pharyngeal injection of distilled water. Animals were anesthetized with pentobarbital (40 mg/kg, i.p.), and then fixed in the supine position on a heated pad. A catheter was inserted through the mouth, with its tip placed into the pharynx. The trachea of the animal was cannulated to maintain respiration. Distilled water was applied to the pharyngolaryngeal region twice, at a flow rate of 10 μl/s for 10 s, at intervals of 3 min. The swallowing movement was identified using the electromyographic activity Power lab (AD Instruments, Nagoya, Japan) and visual observation of the characteristic laryngeal movement. The number of swallows was counted for 10 s after the injection of distilled water, and the mean of the two measurements was expressed. The latency to swallowing onset, which was defined as the time required to elicit the first swallow from the onset of stimulation, was analyzed.

### Analysis of laryngeal residue

We examined the area of the larynx mucosae, stained with pyocyanin blue, to determine whether penetration or aspiration had occurred. At 6 days, the water bottle was changed to a 0.025% pyoktanin aqueous solution (Honzo co. Ltd. Nagoya, Japan) after 16 h water restriction, and animals were allowed to drink freely for 24 h. At 7 days, the animals were sacrificed, and the larynx and trachea were harvested from each group. We measured the percentage of stained area surrounding the epiglottis, aryepiglottic fold and interarytenoid fold in the axial direction. Photographs of the tissues were captured for the larynx and trachea, and the stained area was measured using ImageJ software (http://rsb.info.nih.gov/ij/). Dividing the images into three primary colors, the white area in the image obtained was quantified by subtracting red from blue and excluding green.

### Histomorphological analysis

At 7 days after surgery, nerve-injured segments were harvested and fixed with 2.5% glutaraldehyde (TAAB Laboratories Equipment Ltd., Reading, Berkshire, United Kingdom) overnight at 4 °C. The nerve segments were subsequently fixed in 2% osmium tetroxide (OsO_4_; TAAB Laboratories Equipment Ltd.) for 2 h, separately dehydrated in an ethanol gradient (50%, 70%, 80%, 90%, 95% and 100%), and treated in a gradient of EPON812 (33%, 50%, 66% and 100%; TAAB Laboratories Equipment Ltd.) in propylene oxide (Nacalai Tesque, Inc., Kyoto, Japan). Tissues were embedded in EPON812 in a 60 °C oven for 48 h. Semi-thin sections (200 μm) were cut vertically with an ultramicrotome (Ultracut S; Leica Microsystems, Wetzlar, Germany), stained with 1% toluidine blue solution, and examined under a light microscope (BZ9000; Keyence). The density of the myelinated fibers (fibers/1000 mm^2^) was analyzed in five non-overlapping visual fields per specimen. Ultrathin sections (70–80 nm) were cut with an ultramicrotome. We chose axons exhibiting an equivalent diameter and evaluated the G-ratio as the ratio of the inner axonal diameter to the total outer diameter. The stained samples were observed under TEM (JEM-1400EX; JEOL Ltd., Tokyo. Japan). We randomly selected five separate fields per slice for analysis.

### Immunohistochemical analysis

Rats were deeply anesthetized before undergoing intracardiac perfusion with 4% paraformaldehyde. The SLNs were isolated and embedded in OCT compound (Sakura Finetek, Tokyo, Japan), and 20 μm sagittal sections were generated with a cryostat (Leica CM3050S, Leica Biosystems, Denver, CO). The sections were permeabilized with 0.1% Triton X-100 in phosphate buffered saline for 20 min, blocked with 5% bovine serum for 30 min and incubated overnight with the following primary antibodies: mouse anti-rat CD31 (1:40, 550300 BD Pharmingen), rabbit anti-CD206 (1:1000, ab64693, Abcam) and mouse anti-CD11b (1:1000, ab33827, Abcam). The following secondary antibodies were used: anti-mouse IgG Alexa Fluor 488 and anti-rat IgG Alexa Fluor 647. After counterstaining with 4',6-diamidino-2-phenylindole (DAPI, Sigma-Aldrich), tissue images were observed through a universal fluorescence microscope (BZ9000; Keyence Co., Osaka, Japan).

### Tissue preparation and qRT-PCR

Cervical dislocation was used to kill the animals at 1, 3 and 7 days after injury. The SLNs were harvested from animals in different groups and stored at -80 °C. Total RNA was isolated from the tissues using TRIzol reagent (Invitrogen, Carlsbad, California) according to the manufacturer’s protocol. A spectrophotometer was used to quantify total RNA levels, and RNA integrity was checked on 1% agarose gels. Reverse transcription reactions were performed with Superscript IV reverse transcriptase (Invitrogen, Carlsbad, California) using 0.1 μg total RNA in a 25 μl total reaction volume. The quantitative real-time polymerase chain reaction (qRT-PCR) was performed using THUNDERBIRD SYBR qPCR Mix (Toyobo, Osaka, Japan) and the Mx3000P QPCR System (Agilent Technologies, Tokyo, Japan). The specific primers were designed using Primer3 (S1 Table). All results are normalized to glyceraldehyde 3-phosphate dehydrogenase (GAPDH).

### Statistical analyses

Statistical analyses were performed using SPSS for Windows, version 19.0 (IBM, New York, USA). An unpaired two-tailed Student’s t test was used when comparing two groups. To analyze three or more independent groups, we used a one-way analysis of variance (ANOVA), followed Tukey’s *post hoc* test. Differences were considered statistically significant at *p* < 0.05.

## Results

### A novel visceral nerve lesion model with dysphagia in the rat was established, and SHED-CM was found to improve dysphagia in this model

**Fig 1:**
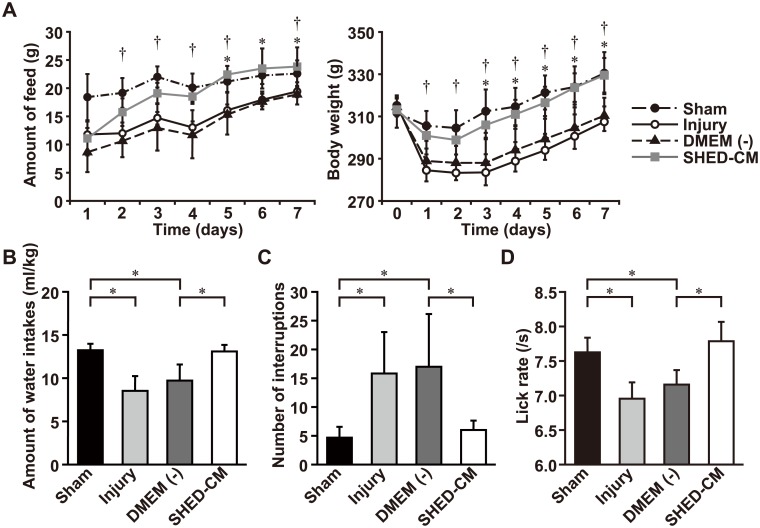
A novel model with dysphagia in the rat was established, and SHED-CM was found to improve dysphagia. (A) Graph showing food intake and body-weight change after SLN injury. n = 6 per group. † *p* < 0.05, sham group versus injury group; * *p* < 0.05, DMEM versus SHED-CM group. Results are presented as mean ± SEM. (B–D) Analysis of swallowing behavior using non-radiographic video recording for 2 min. (B) Measurement of the volume of water intake per body-weight (ml/kg). (C) Measurement of interruptions during swallowing behavior. (D) Measurement of lick rate for 2 min. n = 6 per group. * *p* < 0.05. Results are presented as mean ± SEM.

To elucidate the influence of the nerve lesion, we first measured food intake and body-weight change in a novel animal model of bilateral SLN injury for 7 days. The food intake and body-weights of the rats were significantly reduced after the SLN injury (Fig 1A). Systemic delivery of the SHED-CM resulted in significantly less weight loss compared to the Dulbecco’s Modified Eagles’ Medium (DMEM (-)) group. Similarly, food intake in the SHED-CM group was increased compared to the DMEM (-) group.

To determine whether the SLN lesion could affect swallowing behavior in the rat, we measured the volume of water intake, the number of swallowing interruptions, and the lick rate using 2 min-long video recordings (S1 Video). We counted the number of interruptions, defined by both the number of the head movements of the animal when in drinking position and the number of failures to touch the tip of the water bottle when licking. Licking is the primary way of ingesting liquids and consists in pressing the tongue against the liquid, defined as the oral stage of swallowing [20, 21, 24]. Lick rate indicates the rate of ingestion of liquid into the oral cavity of the rat [24]. The water intake per body weight (ml/kg) decreased in the injury group compared to the sham group (Fig 1B). Following the SLN lesion, the number of interruptions increased and the lick rate was reduced (Fig 1C and 1D). Systemic delivery of SHED-CM significantly reduced the number of interruptions, and increased the lick rate and water intake compared with the DMEM (-) group. These results demonstrate that the SLN lesion caused dysphagia in the oral stage of swallowing in the rat, and SHED-CM improved dysphagia in this stage.

### SHED-CM protects the swallowing reflex

**Fig 2:**
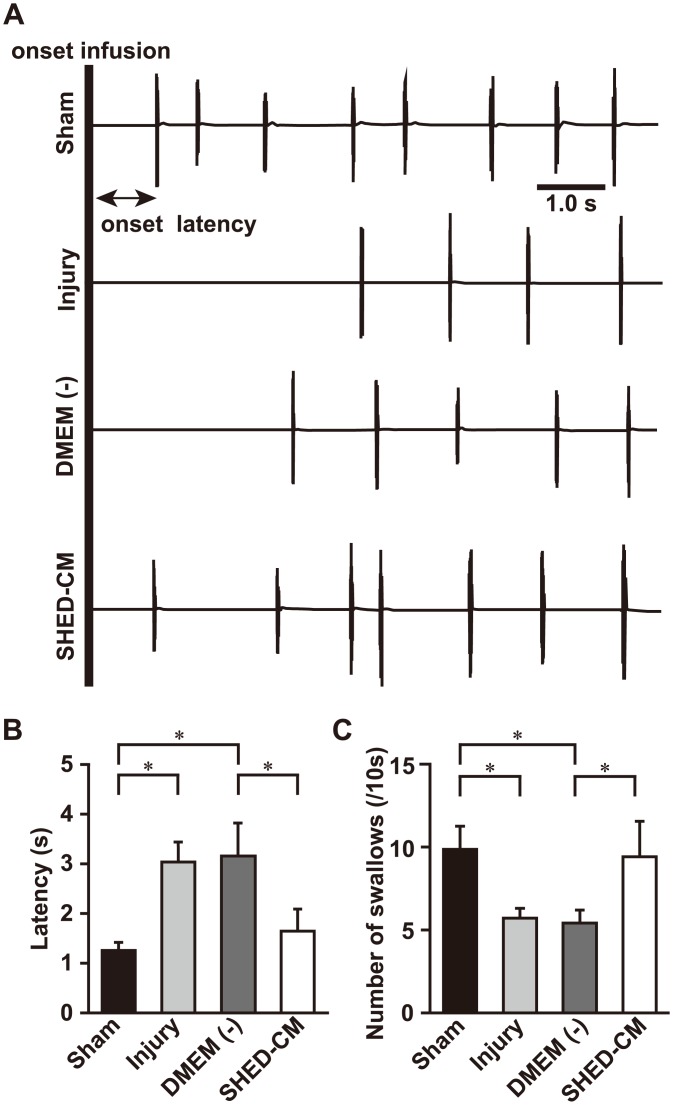
Effects of SHED-CM administration on swallowing initiation. (A) Representative electromyographic recordings from the mylohyoid muscle during swallowing. Measurement of the mean number of swallows (B) and the onset latency to the first swallow (C). SLN injury affects the number of swallows and the latency to swallow. SHED-CM improves the number of swallows and the latency to swallow relative to DMEM (-). n = 6 per group. *p < 0.05. Results are presented as mean ± SEM.

Next, we investigated whether SHED-CM promotes functional recovery after SLN injury. Fig 2A shows examples of electromyographic activity recordings from the mylohyoid muscle during distilled water administration into the pharyngeal region. The mean number of swallows was significantly reduced in the injury group compared to the sham group (Fig 2B). The latency to the first swallow was significantly extended in the injury group compared to the sham group (Fig 2C). These results show that the volume of water required to evoke the swallowing reflex was increased after SLN injury in the rat. Administration of the SHED-CM significantly increased the number of swallows, and shortened the latency to the first swallow compared with DMEM (-) (Fig 2B and 2C). A comparison of the swallowing reflex among the SHED-CM group and DMEM (-) group showed that the administration of SHED-CM improved the swallowing reflex and enhanced functional recovery of the SLN. There were no significant differences between the sham and SHED-CM groups with regard to the mean number of swallows and the latency to the first swallow.

### SHED-CM reduces pharyngeal residue

**Fig 3:**
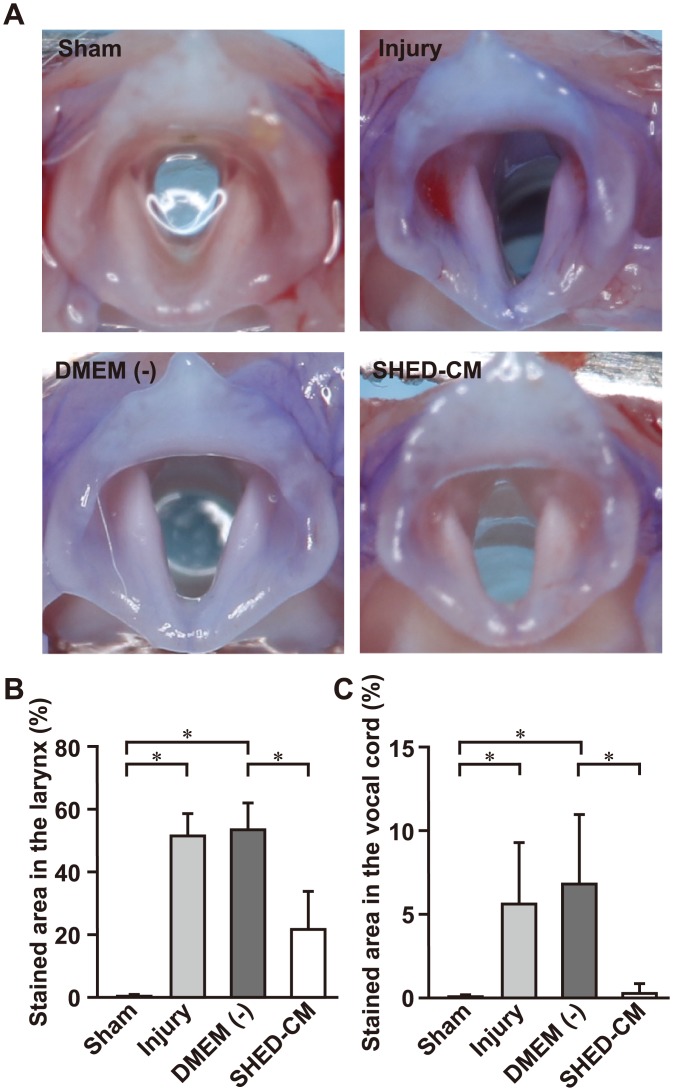
Quantification of the extent of staining in the larynx. (A) Representative image of the larynx in each group, at 7 days after SLN injury. Analysis of the stained area in the larynx (B) and in the vocal cord (C). The stained area in the larynx and vocal cord increases after SLN injury. Treatment with SHED-CM significantly reduces the stained area in the larynx and vocal cord relative to DMEM (-). n = 6 per group. *p < 0.05. Results are presented as mean ± SEM.

To determine whether the SLN lesion affected the pharyngeal stage of swallowing, we investigated the laryngeal residue in the rat larynx. It is well known that the amount of laryngeal residue is a predictor of aspiration and penetration in humans [25]. Previous studies revealed that bilateral SLN injury in pigs affected swallow function and increased aspiration incidence [26]. However, the impact of SLN injury in the rat was not reported. Fig 3A shows an example of the larynx, and we measured the percentages of the larynx and vocal cord areas that were stained. The stained areas in the larynx and vocal cord were expanded in the injury group compared to the sham group (Fig 3B). Administration of SHED-CM reduced the stained area in the larynx and vocal cord when compared with DMEM (-) (Fig 3C). These results demonstrate that the SLN lesion caused dysphagia in the pharyngeal stage of swallowing in the rat. Moreover, SHED-CM prevented water storage in the pharynx, and might reduce the risk of aspiration and penetration. Staining was not observed in the trachea and lungs in either group.

### SHED-CM promotes axon regeneration after SLN injury

**Fig 4:**
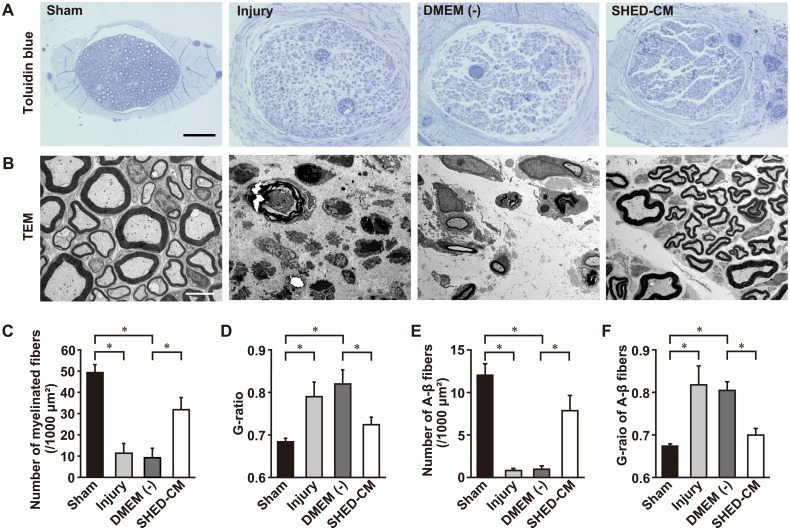
Morphological evaluation of nerve regeneration in the SLN. (A) Toluidine blue staining of semi-thin sections from the middle of the injury segments at 7 days after SLN injury. Scale bar 50 μm. (B) TEM images of ultrathin cross sections of segments from the middle of the injury at 7 days after SLN injury. Scale bar: 5 μm. The injury group shows widespread inflammation and Wallerian degeneration at 7 days after SLN injury. Analysis of myelinated fiber densities (C) and the G-ratios (D). Myelinated fiber densities and the G-ratio in the SHED-CM group significantly improved relative to the DMEM (-) group. Analysis of A-β fiber densities (E) and the G-ratio of the A-β fibers (F). The densities and the G-ratios of the A-β fibers are significantly higher in the SHED-CM group than in the DMEM (-) group. n = 6 per group. *p < 0.05. Results are presented as mean ± SEM.

To examine nerve regeneration, we performed a histological analysis of the SLN in the middle of the damaged area, 7 days after nerve injury. Examples of toluidine blue staining and transmission electron microscopy (TEM) of the SLN cross-sectionally are shown in Fig 4A. The toluidine blue staining showed widespread, severe edema, as well as inflammation and Wallerian degeneration in the injury and DMEM (-) groups. TEM showed that the typical structure of the nerve fibers almost disappeared in the injury and DMEM (-) groups (Fig 4B). In contrast, many nerve myelinated fibers were identified in the SHED-CM group (Fig 4A and 4B). Quantitative TEM analysis showed that the fiber densities in the injury group were significantly lower than in the sham group (Fig 4C). The mean G-ratio, the ratio between the inner and outer diameter of the myelin sheath, was significantly higher in the injury group compared to the sham group. Meanwhile, the SHED-CM group showed higher fiber densities when compared with the DMEM (-) group (Fig 4C). Furthermore, the mean G-ratio showed that the degree of myelination in the SHED-CM group was significantly higher compared to the DMEM (-) group (Fig 4D). There were no significant differences between the sham and SHED-CM groups with regard to the fiber density and G-ratio. In addition, we counted myelinated fibers with an axon diameter of less than 5 μm and measured the G-ratios of these myelinated fibers. In a previous study, myelinated fibers with less than 5 μm of diameter in the SLN were classified as A-β fibers [27]. The number of A-β fibers in the SHED-CM group was significantly increased compared to the DMEM (-) group (Fig 4E). The G-ratio of A-β fibers in the SHED-CM group was higher than in the DMEM (-) group (Fig 4F). These results strongly suggest that SHED-CM promoted functional nerve regeneration following the SLN lesion.

### SHED-CM recruits M2 macrophages at the injury site

**Fig 5:**
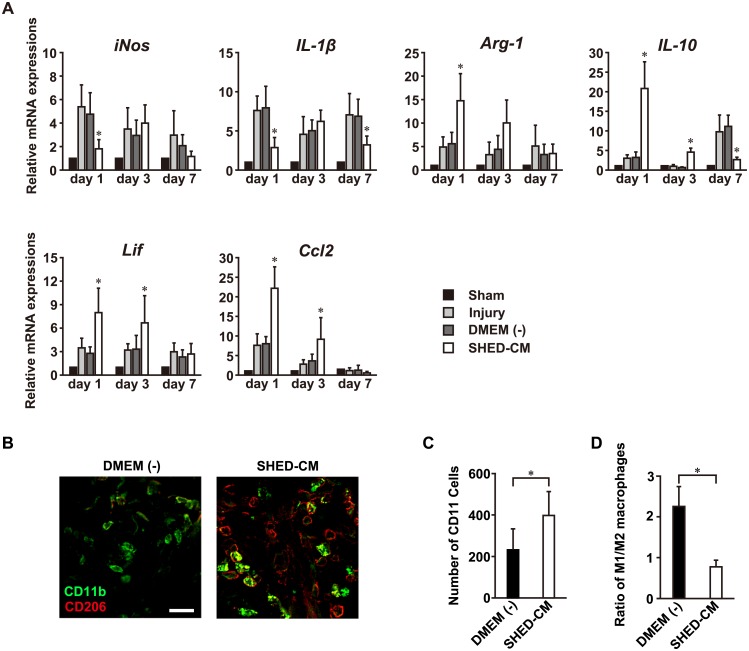
SHED-CM recruits M2 macrophages to the injury site. (A) SHED-CM administration downregulates M1 markers (iNos, IL-1β) and upregulates M2 markers (Arg-1, IL-10). SHED-CM also suppresses the expression of IL-6, and upregulates Lif and Ccl2. Results are expressed relative to the level in the sham-operated model. All results are normalized to GAPDH. n = 6 per group. *p < 0.05. Results are presented as mean ± SEM. (B) Representative images of the immunohistological staining of D206 and CD11b. (C) Quantification of CD11b macrophages at injury site. (D) The proportion of CD11b/CD206-positive macrophages. n = 6 per group. *p < 0.05. Results are presented as mean ± SEM. Scale bar in (B): 20 μm.

We examined the mRNA expression profiles of pro-inflammatory and anti-inflammatory factors in the SLNs (Fig 5A). The administration of SHED-CM markedly suppressed the expression of the pro-inflammatory mediators inducible nitric oxide synthase (iNOS) and interleukin-1 beta (IL-1β), and increased the expression of the anti-inflammatory M2 macrophage markers arginase-1 (Arg-1) and interleukin-10 (IL-10), at 1 day after injury. Leukemia inhibitory factor (Lif) and chemokine C-C motif ligand (Ccl2), also known as monocyte chemoattractant protein-1, contributed both to the attraction of macrophages to the damaged nerve site and to nerve regeneration [28]. These chemokines were also upregulated in the SHED-CM group at earlier time-points after nerve injury. Immunohistochemical analysis showed an accumulation of M2 macrophages at the nerve injury site 3 day after nerve injury (Fig 5B). Quantitative analysis revealed that the number of CD11b macrophages in SHED-CM was increased relative to the DMEM (-) group (Fig 5C). The proportion of CD11b/CD206-positive M2 macrophages was significantly decreased in the SHED-CM group compared to the DMEM (-) group (Fig 5D). These results demonstrate that SHED-CM promotes M2 macrophage recruitment.

### SHED-CM upregulates trophic factors and promotes vascularization at the injury site

**Fig 6:**
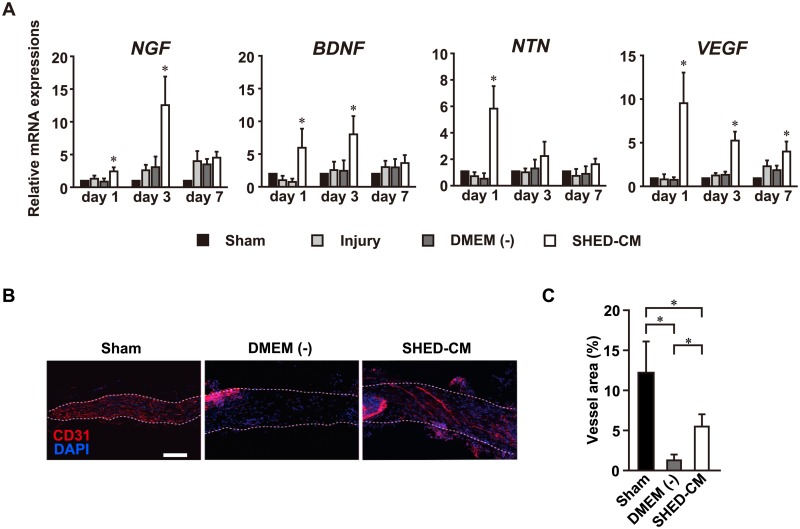
SHED-CM promotes vascularization and recruits M2 macrophages to the injury site. (A) SHED-CM upregulated the expression of multiple trophic factors. Results are expressed relative to the level in the sham-operated model. All results are normalized to GAPDH. n = 6 per group. *p < 0.05. Results are presented as mean ± SEM. (B) Representative images of the immunohistological staining of CD31 in a sagittal section of the SLN at 7 days following the SLN lesion. (C) Quantification of CD31 cells in the injury site. SHED-CM significantly enhanced the area of blood vessels in the nerve lesion site compared to DMEM (-). Scale bar in (B): 100 μm. n = 6 per group. *p < 0.05. Results are presented as mean ± SEM.

Nerve regeneration depends on the expression of neurotrophic factors [29, 30]. Systemic administration of the SHED-CM upregulated levels of neurotrophic factors, including nerve growth factor (NGF), brain-derived neurotrophic factor (BDNF) and neurturin (NTN), and this upregulation peaked at 1 and 3 days after nerve injury (Fig 6A). Furthermore, vascularization in the nerve injury site was investigated. qRT-PCR analysis revealed that the mRNA expression of vascular endothelial growth factor (VEGF) increased at 1, 3, and 7 days after nerve injury compared to the DMEM (-) group (Fig 6A). Immunostaining for the vascular endothelial cell marker CD31 showed that the administration of SHED-CM considerably promoted vascular endothelial cell migration towards the lesion site, as shown in the longitudinally sectioned samples at 7 days after nerve injury (Fig 6B). Quantitative analysis showed that the area of blood vessels in the nerve lesion site was significantly increased in the SHED-CM group compared with the DMEM (-) group (Fig 6C). These results suggest that SHED-CM promotes nerve regeneration via vascularization.

## Discussion

In the present study, we established a novel animal model of visceral nerve lesion that allowed evaluation of rat swallowing function. Our experiments demonstrate that SLN injury in the rat induced weight loss, and reduced food and water intake. Dysphagia caused by the SLN injury also showed that there was a delayed swallowing reflex and pharyngeal storage, indicating dysfunction of the oral and laryngeal stages of swallowing. In addition, our results suggest that administration of SHED-CM following the SLN injury improved swallow function and enhanced nerve regeneration through M2 macrophage polarization and vascularization. Here, we show that our novel animal model of SLN injury can be used as a novel visceral nerve regeneration model, and that SHED-CM may have therapeutic benefits for SLN injury.

Until now, the SLN was widely used in swallowing studies to evoke the swallowing reflex [23, 31]. This study is the first to assess swallowing behavior in rodents following SLN injury. Our study indicated that rats with dysphagia caused by SLNs lesion fail to consume water continuously, and have a reduction in water consumption per unit of time. The reduction in water intake following SLN injury suggests that rats can drink water in small quantities for single swallowing. With regard to oropharyngeal evaluation, previous studies found a correlation between the loss of sensory input from the laryngeal area and the occurrence of penetration and aspiration in humans [32, 33]. Although the relationship between sensory loss in laryngeal area and aspiration in the rat was not unraveled, our data show that the removal of the stimulus from SLN to the larynx causes dysfunction in the oral and laryngeal stages of swallowing.

Moreover, in previous animal studies, swallowing function was analyzed using videofluoroscopic swallow study (VFSS) methods, which were considered the standard gold method for assessing swallowing function clinically [20, 26, 34]. Although VFSS makes it possible to observe, in detail, bolus flow in the oral, oropharyngeal, and esophageal stages of swallowing, the use of VFSS in rats is not easy because of the high swallowing speed of rodents, which is 10 times faster than humans’ [20]. The method of drinking the stain solution, used in our study, was a simple way to compare the degree of laryngeal storage. Thus, our model allowed for the simple evaluation of dysphagia in the pharyngeal stage of swallowing in the rat without VFSS.

A proper inflammatory response, namely macrophage phenotype-switching, is necessary for tissue regeneration after peripheral nerve injury. Resident and infiltrating macrophages and dedifferentiated Schwann cells remove axonal and myelin debris, and create an environment for axonal regeneration [28, 35]. Recent studies have shown that macrophages polarization from the M1 phenotype to the M2 phenotype induced by IL-4 and IL-10 contribute to axonal regeneration [36, 37]. Our findings showed that a single systemic administration of SHED-CM suppresses pro-inflammatory M1 phenotype macrophages, and activates anti-inflammatory M2 phenotype macrophages at the injury site. However, the mechanism behind this transition remains unclear. In recent studies, it was shown that depicting macrophages as having the ability to switch from one phenotype to another phenotype is not quite accurate. Macrophages can change their functional phenotype according to the microenvironment, and multiple cytokine treatment induced multiple functional phenotypes of macrophages [38].

The gene expression pattern in this study showed that SHED-CM induced striking changes in many parameters. In the damaged peripheral nerve, a pro-inflammatory immune response is triggered, with Ccl-2 and Lif regulating macrophage recruitment [39, 40]. These factors are rapidly produced by Schwann cells, and infiltrating macrophages produce a chemokine that stimulate the recruitment of even more macrophages [28]. Additionally, large numbers of macrophages in Wallerian degeneration in peripheral nerve system are mostly recruited from bone marrow [35]. Our data show that the administration of SHED-CM strongly upregulated Ccl-2 and Lif, resulting in a strong attraction of macrophages to the injury site. Although it was difficult to completely distinguish hematogenous macrophages from residential macrophages *in vivo*, the results imply that SHED-CM recruited many hematogenous M2 polarized macrophages, and that they infiltrated into the damaged SLN. Moreover, recent studies showed that neurotrophic factors, including NGF, BDNF and NTN, are important for neuron growth and/or survival [30, 41, 42]. The upregulation of these factors also indicates that SHED-CM promotes tissue repair at the injury site.

Angiogenesis is a crucial process for tissue regeneration. In peripheral nerve regeneration processes, Bungner’s bands, which guide regenerating axons, are formed when axons grow back to their target site following Wallerian degeneration [28]. New blood vessels induced by VEGF-A, which is produced by macrophages under hypoxic conditions within the nerve injury site, allow Schwann cells to migrate and contribute to the formation of Bungner’s bands [43]. Previous *in vitro* studies showed that SHED-CM enhances Schwann cell proliferation and migration, and promotes tube formation by human umbilical vein endothelial cells [17]. hDPSC had the high angiogenic differentiation capabilities [44], and CM from hDPSCs also promotes endothelial cell proliferation and growth [45]. Moreover, microvesicles derived from M2 macrophages promoted nerve regeneration through proliferation and migration of Schwann cells [46]. Taken together, our results, plus the available evidence in the literature, suggest that SHED-CM promotes Bungner’s band formation via new blood vessel formation that allows Schwann cells to migrate.

The VEGF contained in SHED-CM can be considered as a therapeutic candidate. However, use of the VEGF requires for appreciate concentration, timing, and spatial distribution owing to formation of vascular abnormalities [47]. Administration of high doses VEGF without other trophic factors induced fragile and unstable vessels [48] [49]. Moreover, in a clinical trial, intracoronary and intravenous infusion of VEGF did not lead to significant therapeutic benefits [50]. Therefore, we hypothesize that part of the therapeutic effect of SHED-CM is due to factors related to the conversion of M1 macrophages to M2 macrophages, such as IL-4, IL-10, Ccl-2, and the secreted ectodomain of sialic acid binding Ig-like lectin-9 (sSiglec-9). Administration of IL-4 or IL-10 was shown to modulate the ratio of M1 and M2 macrophages [36, 37]. However, the amount of these factors used in the study was higher than the amount present in SHED-CM [18]. Furthermore, although administration of high doses of Ccl-2 and sSiglec-9 (two molecules found in SHED-CM) elicited the same effect as SHED-CM in functional nerve recovery, when these factors are administered at concentration similar to those found in SHED-CM, they elicit insufficient improvement [51]. Additional studies will be necessary to further investigate therapeutic factors in SHED-CM.

In this study, we developed a novel animal model of SLN injury that showed dysphagia in the oral and laryngeal stages of swallowing in the rat. Administration of SHED-CM improved functional recovery in our model, an effect that likely occurs through two mechanisms: macrophage polarization and vascularization. Our study suggests that SHED-CM may provide therapeutic benefits for patients with SLN injury.

## Supporting information

S1 Table Primer sequences used for the qRT-PCR. (DOCX)

S1 Video Comparison of swallowing behavior in video recordings. (MP4)
