# Supplementary material for: Dental pulp-derived stem cell conditioned medium to regenerate peripheral nerves in a novel animal model of dysphagia
Source: PLoS One. 2018 Dec 11;13(12):e0208938. doi: 10.1371/journal.pone.0208938 (PMC6289419; doi:10.1371/journal.pone.0208938)
Supplement: S1 Table — (DOCX) [file pone.0208938.s001.docx]

**S1 Table. Primer sequences used for the qRT-PCR.**

|  |  |  |
| --- | --- | --- |
| Gene | Sequence (forward 5'-3') | Sequence (reverse 5'-3') |
| iNos | F: GCAAGCCCTCACCTACTCCT | R: ACCTCTGCCTGTGCGTCTCT |
|  |  |  |
| IL-1β | F: CATAAGCCAACAAGTGGTATTCTCC | R: GGGTGTGCCGTCTTTCATC |
|  |  |  |
| Arg-1 | F: CACCTGAGTTTTGATGTTGATGG | R: TCCTGAAAGTAGCCCTGTCTTGT |
|  |  |  |
| IL-10 | F: ACTGCAGGACTTTAAGGGTTACTTG | R: GCCTGGGGCATCACTTCTAC |
|  |  |  |
| IL-6 | F: GCCTTCTTGGGACTGATGTTG | R: GGTCTGTTGTGGGTGGTATCCT |
|  |  |  |
| Lif | F: GGTCTTGGCCACAGGGATT | R: TTACAGGGGTGATGGGAAGG |
|  |  |  |
| Ccl2 | F: ACCAGAACCAAGTGAGATCAGAA | R: GGTTGTGGAAAGAGAGTGGATG |
|  |  |  |
| NGF | F: CATCCACCCACCCAGTCTTC | R: ATGTCCGTGGCTGTGGTCTT |
|  |  |  |
| BDNF | F: CGTCCCTGGCTGACACTTT | R: TTTCTCCGCTCTGAACAAGG |
|  |  |  |
| VEGF | F: ACCAAAGCCAGCACATAGGA | R: GGGGCATTAACTGCATCTGG |
|  |  |  |
| GDNF | F: ACCAAAGCCAGCACATAGGA | R: TTTCTCCGCTCTGAACAAGG |
